# Carbohydrate Sulfotransferase 4 Inhibits the Progression of Hepatitis B Virus-Related Hepatocellular Carcinoma and Is a Potential Prognostic Marker in Several Tumors

**DOI:** 10.3389/fonc.2020.554331

**Published:** 2020-10-15

**Authors:** Longshan Zhang, Yao Fan, Xiaoqing Wang, Mi Yang, XiXi Wu, Weiqiang Huang, Jin Lan, Liwei Liao, Wenqi Huang, Lu Yuan, Hua Pan, Yuting Wu, Longhua Chen, Jian Guan

**Affiliations:** ^1^Department of Radiation Oncology, Nanfang Hospital, Southern Medical University, Guangzhou, China; ^2^Department of Oncology, The First Hospital of Hebei Medical University, Shijiazhuang, China; ^3^Department of General Surgery, The Third Affiliated Hospital of Southern Medical University, Guangzhou, China

**Keywords:** HBV-related hepatocellular carcinoma, CHST4, prognostic biomarker, expression profiling, competing endogenous RNA

## Abstract

Carbohydrate sulfotransferase 4 (CHST4) plays an important role in lymphocyte homing and is abnormally expressed in several cancer types; however, its precise function in tumor development and progression is unknown. Here we confirm that CHST4 is aberrantly expressed in various tumor subtypes. In particular, we found that CHST4 expression was downregulated in hepatitis B virus-related hepatocellular carcinoma (HBV-HCC) tumors compared to paired normal tissue. We also showed that CHST4 overexpression inhibited the proliferation and metastasis of HCC cells *in vitro*. Clinically, CHST4 was identified as an independent prognostic factor for HBV-HCC patients. We further illuminated the anti-tumor role and mechanism of CHST4 in HBV-HCC by constructing a FENDRR–miR-10b-5p–CHST4 competing endogenous RNA network. We found that downregulation of CHST4 expression may promote HBV expression and regulate ribonucleoprotein complex biogenesis to promote malignant behaviors in HBV-HCC. CHST4 may also recruit CD4+ T cells, macrophages, dendritic cells, and neutrophils into the tumor microenvironment to inhibit the progression of HBV-HCC. Overall, our findings suggest that CHST4 acts as a tumor suppressor in HCC-HBV and represents a potential diagnostic and therapeutic target.

## Introduction

Hepatocellular carcinoma (HCC) is the fifth most common cancer and the third leading cause of cancer deaths worldwide (1–3). Although there has been considerable progress in the treatment of HCC, patient prognosis remains bleak (4). Chronic hepatitis B virus (HBV) infection is a major risk factor for HCC (5) and is responsible for about 50% of HCC cases and almost all childhood liver cancer (6). Indeed, in China, most HCC is related to HBV infection (7–9). Many biomarkers have been used to monitor at-risk populations, such as alpha-fetoprotein L3 (AFP-L3), des gamma carboxyprothrombin (DCP), glypican 3 (GPC3), and Golgi membrane protein 73 (GP73), yet these serum markers are not elevated in all HCC subtypes (10, 11). Therefore, effective and reliable biomarkers for the early detection of HCC are in high demand.

According to the Human Protein Atlas, CHST4 (carbohydrate sulfotransferase 4, GlcNAc6ST2) is expressed in the gallbladder, fallopian tube, liver, and pancreas. CHST4 is a member of the GlcNAc6ST family of proteins and catalyzes the transfer of sulfate to position 6 of non-reducing GlcNAc residues within mucin-associated glycans that ultimately serve as L-selectin ligands (12, 13). These L-selectin ligands are present in high endothelial cells (HEVs) and contribute to lymphocyte homing (14–16). Although previous researches showed that CHST4 was specifically expressed in HEVs (17), follow-up studies have revealed that CHST4 is abnormally expressed in a variety of solid tumors. For example, CHST4 is highly expressed in mucinous adenocarcinomas (18, 19), where it adds sulfate to extended core 1- and core 2-based-glycans (20). Additionally, in those with early-stage uterine corpus and cervical cancer, serum CHST4 expression was higher than that of cancer antigen 125 (CA125) and squamous cell carcinoma antigen (SCC) (21). CHST4 expression is also elevated in gastric cancer tissues (22), urothelial bladder carcinoma (23), cholangiolocellular carcinoma (24), and *Opisthorchis viverrini* (OV)-related intrahepatic cholangiocarcinoma (25), and the CHST4 promoter is hypermethylated in HBV-HCC (26). In gliomas, CHST4 was shown to have a high frequency of mutant alleles and amplifications and was related to poor prognosis (27). Overall, the current evidence suggests that CHST4 is a cancer-related enzyme and is a potential biomarker in some tumor subtypes. However, the specific functions and roles of CHST4 in tumor progression are largely unknown.

Although CHST4 has been implicated in HBV-HCC (26), its precise function and prognostic value remain unclear. Therefore, this study aimed to clarify the function and clinical value of CHST4 in cancer by performing expression profiling in mice and human tumors. The function of CHST4 in HBV-HCC was also analyzed both *in vitro* and *in vivo*.

## Materials and Methods

### Mouse Tissues

Four male BALB/c mice (4 weeks old) were obtained from the Animal Science Centre Laboratory of Southern University (Guangzhou). All animals were housed in a specific pathogen-free (SPF)-class and temperature-controlled sterile room. All mice were sacrificed by cervical dislocation, and different organs and tissues were obtained. All collected tissues were stored at −80°C for subsequent quantitative real-time-PCR (qRT-PCR) and immunohistochemistry examination. The BALB/c mice were obtained from the Animal Science Centre Laboratory of Southern University (Guangzhou) with the approval of the Institutional Animal Care Committee of Southern Medical University.

### Clinical Specimens and Patients

Eight paired tumors and normal tissues were collected from 76 patients who underwent surgical resection at the Nanfang Hospital (Guangzhou, China) from January 2019 to October 2019, with over three specimens collected for each patient. Meanwhile, 40 pairs of freshly resected HCC and adjacent non-neoplastic liver tissues were collected from patients who had undergone hepatectomies for the curative treatment.

All patients had not received chemotherapy, radiotherapy, multiple operation, and biological immunotherapy before surgery. HCC patients were HBV positive, while recurrent HCC and incomplete follow-up data were excluded. The BCLC staging system was used for staging. All collected tissues were stored at -80°C until needed. All patients provided informed consent, and the study was approved by the Institutional Review Board of Nanfang Hospital.

### Immunohistochemistry

Four-micrometer-thick sections from each sample were cut, deparaffinized using xylene, hydrated through graded alcohol, and incubated with 3% hydrogen peroxide for endogenous peroxidase blockade, and processed for antigen retrieval by microwave heating for 15 min in 1 mM Tris–EDTA buffer (pH 8.0). The primary anti-CHST4 antibody for mice (ARP35787_T100, Aviva Systems Biology) was diluted 1:50 in PBS containing 1% BSA, and the anti-CHST4 antibody for humans (66623-1-Ig, Proteintech) was diluted 1:200, prior to incubation with the sections (overnight at 4°C). As a negative control, a section on each slide was incubated with rabbit serum (provided by Aviva Systems Biology) for mice and mouse monoclonal IgG (B900620, Proteintech) for humans. Subsequently, the sections were incubated with horseradish-peroxidase-conjugated anti-rabbit secondary antibody (DakoCytomation, Glostrup, Denmark) for 1 h at 37°C and then developed with diaminobenzidine. All sections were counterstained with hematoxylin, visualized under a microscope (Olympus, Japan; four images per section). Two pathologists, who were blind to clinical data, calculated CHST4 expression as the sum of the percent positivity of stained tumor cells and the staining intensity (28), and they independently scored CHST4 expression in human tumors as negative, low, moderate, or high. ImageJ software (National Institutes of Health, Bethesda, MD, United States) was used for quantitative analysis of the immunohistochemical staining (29).

### qRT-PCR

Total RNA was isolated from tissues or cells using TRIzol (Invitrogen, United States) and reverse transcribed using the PrimeScript RT Reagent Kit (Perfect Real Time, Takara). qRT-PCR was performed with TB Green^TM^ Premix Ex Taq^TM^ II (Tli RNaseH Plus, Takara) using the ABI 7500 fast Real-Time PCR System (Applied Biosystems 7500 Fast), as previously described (30). The primer sequences used for CHST4 (human) were: forward: 5′-CCTGCTGTTTCTGGTTTCCCA-3′, reverse: 5′-TGCCCCACAAAAGAAGAGCC-3′; and those for CHST4 (mice) were: forward: 5′-TCCATACTAACGCCAGGAACG-3′, reverse: 5′- TGGTGACTAAGGCTGGAACC-3′. The relative CHST4 level was calculated using the comparative Ct method (ΔΔCt), and normalized to the reference gene, GAPDH.

### Cell Lines and Cell Culture

All cell lines were obtained from the Shanghai Institute for Biological Sciences (Shanghai, China). The human hepatoma cell lines (HCCL-M3, MHCC-97h, Huh7, SMMC-7721, HepG2, and HepG1), and the immortalized normal hepatocyte cell line (LO2) were cultured in high glucose DMEM (Biological Industries, Cronwell, CT, United States) supplemented with 10% fetal bovine serum (FBS; Biological Industries, Cronwell, CT, United States). All media were supplemented with 100 U/ml penicillin and 100 μg/ml streptomycin (GibcoTM, Thermo Fisher Scientific, Waltham, MA, United States) and incubated at 37°C in a humidified chamber containing 5% CO_2_.

### Plasmids and siRNA Transfection

Cells were transfected with CHST4 plasmid (GeneCopoeia, United States) or human siRNA1, siRNA2, and siRNA3 sequences (sense: 5′-CGTCAGATCTGAACAAGAA-3′; sense: 5′-GGAGGACCAACCCTACTAT-3′; sense: 5′-GGAGATCTCATGATTGACA-3′; synthesized by RiboBio) by Lipofectamine^®^2000, as per manufacturer’s instructions (Invitrogen, Carlsbad, CA, United States). RNA and protein were collected at 48 and 72 h after transfection, and CHST4 expression was detected by qRT-PCR and Western blot to verify the transfection efficiency, as previously described (31).

### Western Blotting

Western blotting was performed according to standard methods, and antibodies were used against CHST4 (1:500, 66623-1-Ig, Proteintech) and GAPDH (1:1,000, 60004-1-Ig, Proteintech).

### Cell Proliferation and Migration Assay

Cells were seeded into 96-well plates and incubated at 37°C. A cell counting kit-8 (CCK-8; Dojindo Molecular Technologies, Kumamoto, Japan) was used to detect viable cells over 7 days. Briefly, 10 μl of CCK-8 solution was added, and the absorbance at 450 nm was measured after 1 h (three duplicate wells/sample). Additionally, *in vitro* Transwell assays were performed to assess cell migration ability. At 24 h after siRNA or plasmid transfection, 5 × 10^4^ cells (cultured with serum-free medium) were placed in the upper insert dish of a 24-well plate chamber filled with DMEM containing 10% FBS (Corning, United States). After incubation for an appropriate period of time at 37°C, the cells were fixed with 100% methanol for 40 min and stained with crystal violet solution for 15 min. Migrated cells were photographed and counted in five random regions with an inverted microscope.

### Bioinformatics Analysis

The chromosome location and gene structure of *CHST4* were analyzed by GeneCards^1^ and Uniprot^2^. The protein sequences of CHST4 from human and other species were compared using DNAMAN software (Lynnon Biosoft, United States). The expression profiles of CHST4 in various human normal and cancer tissues were examined using the TIMER platform^3^. The CHST4 mRNA expression and copy number variation (CNV) in human cancer cell lines were analyzed using the CCLE database^4^. CHST4 mutations in human tumors were evaluated via the cBioPortal database^5^. The clinical prognosis of CHST4 in human tissues was analyzed using the GEPIA database^6^, which is based on The Cancer Genome Atlas (TCGA).

DIANA, miRDB, mirDIP, and miRwalk databases were used to predict microRNAs (miRNAs) that target CHST4, and the DIANA LncBase V2 database was used to predict long non-coding RNAs (lncRNAs). The competing endogenous RNA (ceRNA) network was constructed using the Cytoscape software (28). The heatmaps of differentially expressed miRNAs and lncRNAs were produced using GraphPad Prism (v8). In addition, the Gene Ontology (GO) enrichment analysis and the Kyoto Encyclopedia of Genes and Genomes (KEGG) pathways were generated by ImageGP^7^.

We screened for genes that interact with CHST4 using the STRING database^8^. The expressions of CHST4, miRNAs, and lncRNAs were obtained from TCGA^9^. CHST4 gene expression and corresponding clinical data were obtained from the GSE14520 dataset of HBV-HCC and downloaded from the Gene Expression Omnibus (GEO) database^10^. The X-tile program was used to select the cut-off value of CHST4 expression in GSE14520. To investigate the difference of biological functions and pathways between high and low expression groups of CHST4 in HBV-HCC survival, gene set enrichment analysis (GSEA) was used to investigate potential mechanisms in the Molecular Signatures Database (MSigDB) of c2 (c2.cp.kegg.v7.1.symbols) and c5 (c5.all.v7.1.symbols) (32). False discovery rate (FDR) <0.25 and the nominal *P*-value < 0.05 were considered statistically significant. We examined the correlation between the expression level of CHST4 and other genes using two-side Pearson correlation coefficients and the z-test (33). The “cor” function of the R software (34) (R version 3.5.1) was used in this study (| Pearson correlation coefficient| > 0.5 and *P* < 0.01). The degree of immune infiltration in HBV-HCC was evaluated by the TIMER platform (TIMER, QUANTISEQ, MCPCOUNTER, and EPIC databases) (35, 36) and R-ssGSEA algorithm (37).

### Statistical Analyses

All statistical analyses were carried out using SPSS version 22.0 (IBM, United States). All results are presented as mean ± SD. All experiments were performed in triplicate unless otherwise specified. The comparison between the two groups was performed using the Student’s t-test. The associations between the clinicopathological characteristics of the patients and CHST4 expression were analyzed using the Chi-squared test. Univariate and multivariate Cox regression analyses were used to evaluate survival data. The receiver-operating characteristic (ROC) curve was plotted by SPSS, and the area under the ROC curve (AUC) was used for the predicted value. In all tests, a two-tailed *P* < 0.05 was considered statistically significant.

### Safety Statement

For research involving biohazards, correct standard procedures have been carried out.

## Results

### CHST4 Is Highly Conserved in Mammals

**FIGURE 1:**
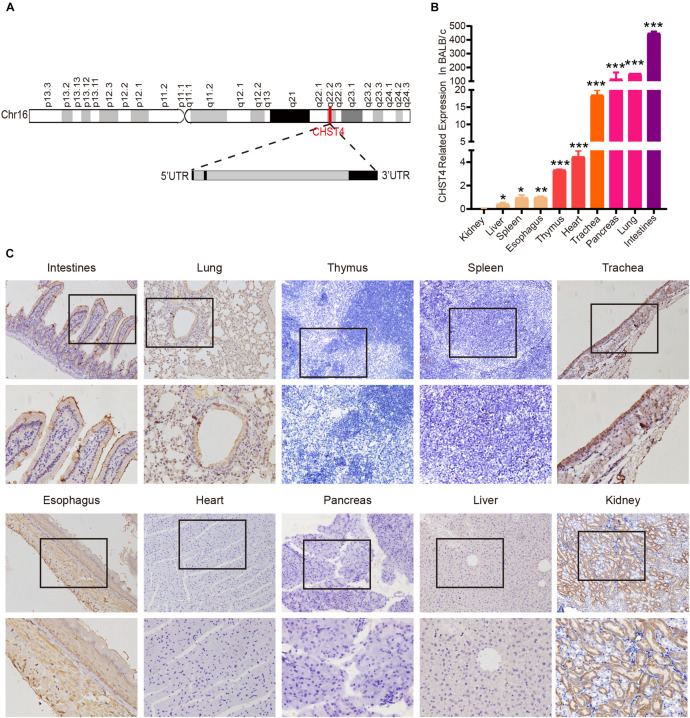
Structure of the carbohydrate sulfotransferase 4 (CHST4) gene and its distribution in BALB/c mice tissues. **(A)** Chromosome location (red line; top) and gene structure of CHST4 (bottom; black boxes represent exons and gray boxes represent introns). **(B)** Relative expression of CHST4 mRNA in various tissues of BALB/c mice by quantitative real-time (qRT)-PCR. GAPDH was used as the loading control. Bars represent the mean ± SD (*n* = 4); *P < 0.05, **P < 0.01, ***P < 0.001 (Student’s *t*-test). All samples were tested in triplicate. **(C)** Immunohistochemical analysis of the expression and distribution of CHST4 protein in BALB/c mice tissues (100×; 400×; four samples in each group).

The *CHST4* gene is located at 16q22.2 and includes a 5′UTR exon, one CDS exon, a 3′UTR exon, and two introns (Figure 1A). Human CHST4 shares 78, 78, 79, 72, and 79% sequence identity to *Bos taurus*, *Capra hircus*, *Felis catus*, *Mus musculus*, and *Sus scrofa* CHST4, respectively, suggesting that CHST4 is highly conserved in mammals (Supplementary Figure S1).

### Distribution of CHST4 in Mice

We used BALB/c mice, the most commonly used laboratory animal, to examine the expression of CHST4 in 10 different tissue types by qRT-PCR. As shown in Figure 1B, mRNA expression of CHST4 was highest in the intestine, followed by the lung and pancreas, then the trachea, and finally, the heart and thymus tissues. Meanwhile, there was little mRNA expression of CHST4 in the kidney, liver, spleen, and esophagus tissues. In terms of protein expression (Figure 1C and Supplementary Figure S2A), low levels of CHST4 were observed in intestinal epithelial cells, and strong expression was found in goblet cells. In the respiratory system, CHST4 was low expressed in the bronchial and alveolar epithelial cells of the lung and moderately expressed in the tracheal epithelial cells. CHST4 was also moderately expressed in the epithelial cells of renal tubules. Esophageal epithelial cells and smooth muscle cells showed low expression. However, there was no protein expression of CHST4 in the thymus, spleen, myocardium, pancreas, or liver. The corresponding negative control is shown in Supplementary Figure S2B.

### CHST4 Expression in Human Tumors

**FIGURE 2:**
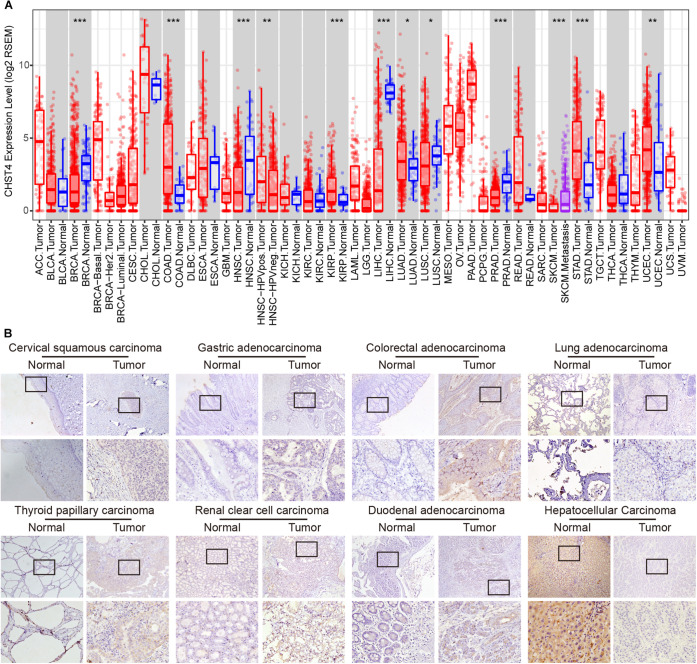
Expression, copy number, and gene alteration of CHST4 in human tumors. **(A)** mRNA expression of CHST4 in 34 types of human tumors, alone or with their paired normal tissues from the TIMER platform. Bars represent mean ± SD; *P < 0.05, **P < 0.01, ***P < 0.001 (Student’s *t*-test).588896101588896101 **(B)** Immunohistochemical analysis of the expression and distribution of CHST4 proteins in human tissues (100×; 400×; more than three samples in each group).

We used the TIMER platform to analyze the expression of CHST4 mRNA in 34 types of human tumors, either alone or with their paired normal tissues. As shown in Figure 2A, CHST4 was highly expressed in cholangiocarcinoma (CHOL), mesothelioma (MESO), and prostate adenocarcinoma (PRAD), and was only lowly expressed in skin cutaneous melanoma (SKCM) and uveal melanoma (UVM). Among the subtypes of breast invasive carcinoma (BRCA), the CHST4 expression in BRCA-basal was significantly higher than in BRCA-Her2 and BRCA-luminal. Interestingly, there was a higher expression level of CHST4 in metastatic SKCM than in non-metastatic SKCM. In head and neck squamous cell carcinoma (HNSCC), CHST4 expression was higher in human papillomavirus (HPV)-positive subtypes than those that were HPV-negative. Five types of human tumors showed higher mRNA expression of CHST4 in the tumor compared to the adjacent normal tissue, including colon adenocarcinoma (COAD), stomach adenocarcinoma (STAD), and kidney renal papillary cell carcinoma (KIRP). Meanwhile, five types of human tumors showed lower CHST4 expression than the adjacent normal tissue, especially BRCA and liver hepatocellular carcinoma (LIHC).

We further detected the protein expression of CHST4 in eight types of human tumors and paired normal tissues by immunohistochemistry. As shown in Figure 2B and Supplementary Figure S3A, the CHST4 was weakly expressed in cervical squamous carcinoma, gastric adenocarcinoma, thyroid papillary carcinoma, renal clear cell carcinoma, and duodenal adenocarcinoma compared to the matched normal tissues. Additionally, CHST4 was strongly expressed in LIHC compared to the paired normal tissues. Moreover, CHST4 was moderately expressed in colorectal adenocarcinoma but not expressed in paired normal tissues. Meanwhile, there was no obvious protein expression of CHST4 in either normal or cancerous tissues of the lung. CHST4 was distributed in the nuclei in LIHC and gastric adenocarcinoma; in all other tissues, it was distributed in the cytoplasm. The corresponding negative control is shown in Supplementary Figure S3B.

We used the Cancer Cell Line Encyclopedia (CCLE) database to explore the role of CHST4 in human tumor cell lines. Both mRNA expression and copy number of CHST4 were highest in the pancreas, colon, lung non-small-cell, and lymphoma Burkitt cell lines (Supplementary Figures S4A,B). We also used the cBioPortal database to analyze CHST4 gene alterations in 32 human tumor types (Supplementary Figure S4C). The change rate of CHST4 (primarily gene amplification) was highest in cholangiocarcinoma, followed by melanoma and uterine cancer (mainly gene mutation).

### Correlation Between CHST4 Expression and Clinical Prognosis

**FIGURE 3:**
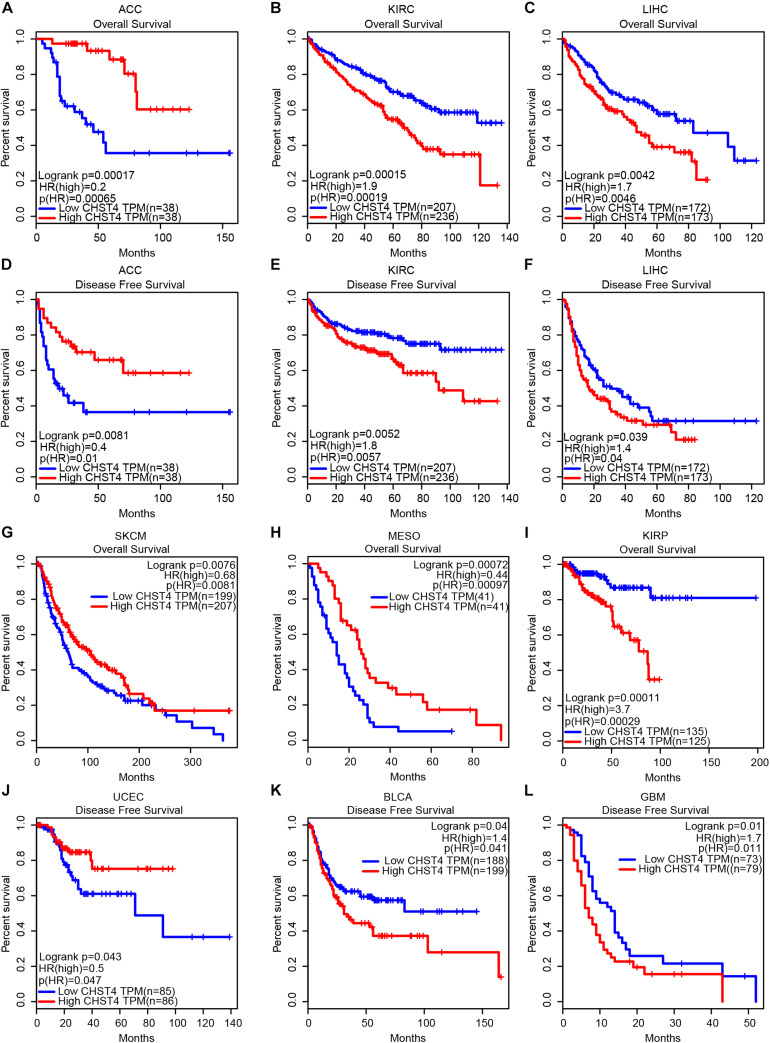
The significance of CHST4 in the clinical prognosis of human tumors. **(A–L)** Kaplan–Meier analysis of the overall survival and disease-free survival in patients with ACC, adrenocortical carcinoma; KIRC, kidney renal clear cell carcinoma; LIHC, liver hepatocellular carcinoma; SKCM, skin cutaneous melanoma; MESO, mesothelioma; KIRP, kidney renal papillary cell carcinoma; UCEC, uterine corpus endometrial carcinoma; BLCA, bladder urothelial carcinoma, and GBM, glioblastoma multiforme using the GEPIA database. The division of CHST4 high and low expression is based on the median (50%) of CHST4 expression in each tumor type.

We used the GEPIA database to analyze the correlation between CHST4 expression and the prognosis of patients with tumors. As shown in Figure 3, in adrenocortical carcinoma (ACC), patients with high CHST4 expression had longer overall survival (OS) and disease-free survival (DFS) than those with low CHST4 expression. However, in kidney renal clear cell carcinoma (KIRC) and LIHC, the opposite was observed: patients with high CHST4 expression had shorter OS and DFS. In SKCM and MESO, patients with high CHST4 expression had longer OS than those with low expression. Additionally, in uterine corpus endometrial carcinoma (UCEC), those with high CHST4 expression had longer DFS than those with low expression. In KIRP, patients with high CHST4 expression had short OS. Finally, in bladder urothelial carcinoma (BLCA) and glioblastoma multiforme (GBM), those with high CHST4 expression had shorter DFS than those with low expression. Therefore, the relationship between CHST4 expression and prognosis varies according to the tumor type.

In HCC, patients with high CHST4 expression had shorter OS and DFS, which suggests that CHST4 plays a tumor-promoting role in HCC. As most HCC is related to HBV in China (7, 8), we sought to determine CHST4 expression in Chinese patients with HBV-HCC using the GEO gene chip. We found that CHST4 expression was lower in tumor tissues than in normal tissues, and HBV-HCC patients with high CHST4 expression had longer OS and DFS. This finding indicates that CHST4 may inhibit the development or progression of HBV-HCC.

### High CHST4 Expression Is Related to Better Prognoses in Patients With HBV-HCC

**FIGURE 4:**
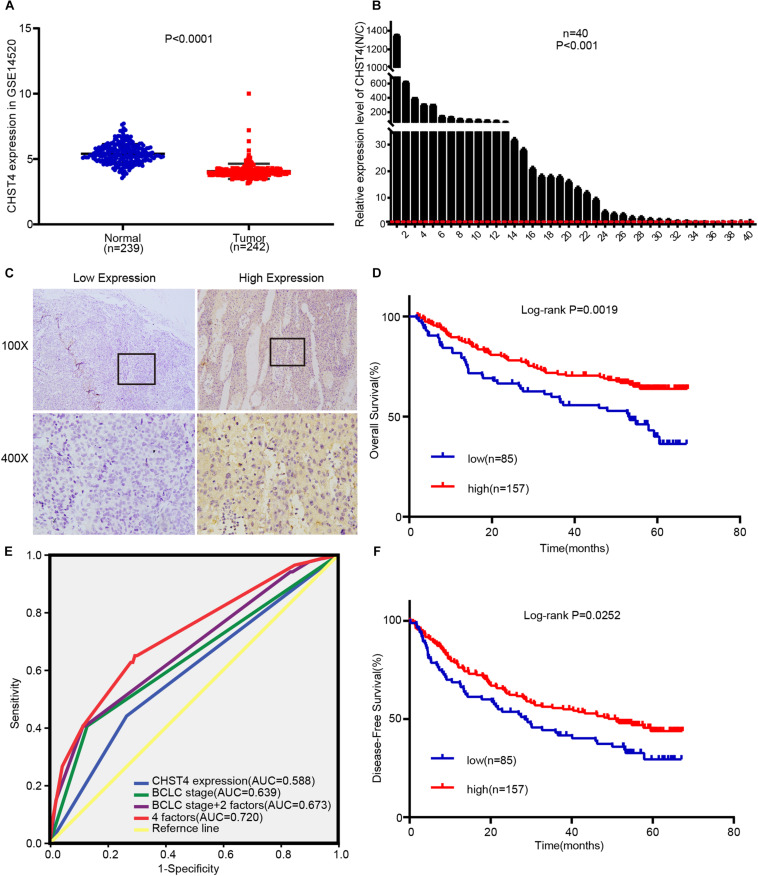
High CHST4 expression is related to better prognoses in patients with hepatitis B virus-related hepatocellular carcinoma (HBV-HCC). **(A)** Expression of CHST4 in patients from the GSE14520 dataset. **(B)** mRNA expression of CHST4 in 40 paired HCC and normal tissue samples (N/C) by qRT-PCR assays. All samples were tested in triplicate. The statistical analysis is paired-samples t-test. Bars represent the mean ± SD. **(C)** Representative staining of low and high CHST4 expression in the tumor cell cytoplasm. **(D,F)**. Kaplan–Meier analysis of overall survival and disease-free survival in those with high CHST4 expression in the GSE14520 dataset versus low expression. **(E)** Receiver-operating characteristic curves (ROC) of the prognostic accuracy of CHST4 expression and clinicopathological risk factors of patients in the GSE14520 dataset. Comparative ROC curves for CHST4 expression, Barcelona Clinic Liver Cancer (BCLC) stage, and BCLC stage with two factors (multinodular and cirrhosis) or with four factors (CHST4 expression, BCLC stage, multinodular, and cirrhosis) are shown (AUC, area under the curve).

After excluding the patients without HBV infection reports and complete survival information from the GSE14520 dataset, we analyzed a total of 242 HBV-HCC tumor tissues and 239 adjacent normal liver tissues. As shown in Figure 4A, CHST4 expression was significantly lower in tumors than in normal tissues (*P* < 0.0001). This finding was verified in our collected HBV-HCC samples (*P* < 0.001) (Figure 4B). However, we found that the CHST4 expression levels varied among different HBV-HCC specimens (Figure 4C). Therefore, we focused on the aberrant expression of CHST4 in tumor tissues.

We used X-tile analysis (38, 39) to divide patients in the GSE14520 dataset into two groups: those with high or low expression of CHST4 (Supplementary Figure S5). We then analyzed the relationship between CHST4 expression and clinicopathological characteristics. As shown in Table 1, strong associations were observed between CHST4 expression and alanine transaminase levels (*P* = 0.026) and TNM staging (*P* = 0.042). However, the expression of CHST4 was not associated with age, gender, tumor size, multinodular, cirrhosis, BCLC stage, or serum AFP (*P* > 0.05).

**TABLE 1:** Correlation between carbohydrate sulfotransferase 4 (CHST4) expression and clinicopathologic characteristics of HBV-HCC patients from the GSE14520 dataset.

	Patient		CHST4				
Variable	No.	%	Low (*n* = 85)	%	High (*n* = 157)	%	*P*-value
Age (years)							0.98
≤50	125	51.7	44	51.8	81	51.6	
>50	117	48.3	41	48.2	76	48.4	
Gender							0.654
Female	31	12.8	12	14.1	19	12.1	
Male	211	87.2	73	85.9	138	87.9	
ALT (U/L)							**0.026**
≤50	142	58.7	58	68.2	84	53.5	
>50	100	41.3	27	31.8	73	46.5	
Tumor size (cm)							0.13
≤5	153	63.2	48	56.5	105	66.9	
>5	88	36.4	36	42.4	52	33.1	
NA	1	0.4	1	1.2	0	0	
Multinodular							0.221
Single	190	78.5	63	74.1	127	80.9	
Multiple	52	21.5	22	25.9	30	19.1	
Cirrhosis							0.096
No	19	7.9	10	11.8	9	5.7	
Yes	223	92.1	75	88.2	148	94.3	
TNM stage							**0.042**
I–II	174	71.9	55	64.7	119	75.8	
III	51	21.1	24	28.2	27	17.2	
N/A	17	7	6	7.1	11	7	
BCLC stage							0.207
0 and A	172	71.1	55	64.7	117	74.5	
B and C	53	21.9	24	28.2	29	18.5	
N/A	17	7	6	7.1	11	7	
Serum AFP (ng/ml)							0.135
≤300	128	52.9	38	44.7	90	57.3	
>300	110	45.5	46	54.1	64	40.8	
N/A	4	1.7	1	1.2	3	1.9	

*HBV, hepatitis B virus; HCC, hepatocellular carcinoma; ALT, alanine transaminase; BCLC, Barcelona Clinic Liver Cancer; AFP, α-fetoprotein; N/A, not available. *P* < 0.05 was considered statistically significant (chi-square test). Bold values means that the results are statistically significant.*

To determine whether CHST4 expression was an independent prognostic factor in HBV-HCC, we performed Cox regression analysis. As shown in Table 2, high expression of CHST4 was associated with a significantly lower risk of death in HBV-HCC patients (*P* = 0.013) compared to those with low CHST4 expression. Moreover, we found that CHST4 expression level could be used to predict survival (*P* = 0.001). We also found that multinodular (*P* = 0.017), cirrhosis (*P* = 0.019), and BCLC stage (*P* = 0.001) were independent prognostic factors for survival in HBV-HCC. When these four parameters were considered together (Figure 4E), the area under the curve (AUC) increased to 0.72 (95% CI 0.651–0.79), which was higher than using the BCLC stage alone (0.639, 95% CI 0.562–0.716) (Supplementary Table S1). We also proved high CHST4 expression correlated with a significantly decreased risk of recurrence in HBV-HCC patients (*P* = 0.026). Furthermore, CHST4 expression (*P* = 0.027), gender (*P* = 0.016), and BCLC stage (*P* = 0.044) were independent prognostic factors for recurrence in HBV-HCC (Table 2). Kaplan–Meier survival curves also demonstrated that patients with higher expression of CHST4 had better OS (*P* = 0.0019) and longer time to recurrence (*P* = 0.0252) (Figures 4D,F). Together, these data suggest that increased CHST4 expression in HBV-HCC correlates with a better prognosis.

**TABLE 2:** Univariate and multivariate analyses of prognostic factors of HBV-HCC patients from the GSE14520 dataset.

	Overall survival			Disease-free survival		
	Univariate analysis	Multivariate analysis		Univariate analysis	Multivariate analysis	
Variables	*P*-value	HR (95% CI)	*P*	*P*-value	HR (95% CI)	*P*-value
Age (years)	0.687			0.657		
≤50						
>50						
Gender	0.094			**0.009**		**0.016**
Female					0.448 (0.233–0.861)	
Male						
ALT (U/L)	0.483			0.06		
≤50						
>50						
Tumor size (cm)	**0.01**		0.994	**0.029**		0.322
≤5		1.002 (0.579–1.735)			1.205 (0.833–1.742)	
>5						
N/A						
Multinodular	**0.025**		**0.017**	0.132		
Single		2.138 (1.142–4.000)				
Multiple						
Cirrhosis	**0.023**		**0.019**	0.074		
No		0.182 (0.044–0.757)				
Yes						
TNM staging	**0.001**		0.168	**0.02**		0.453
I–II		1.691 (0.801–3.569)			0.780 (0.408–1.493)	
III						
N/A						
BCLC stage	**<0.001**		**0.001**	**0.002**		**0.044**
0 and A		3.837 (1.727–1.873)			1.868 (1.017–3.428)	
B and C						
N/A						
Serum AFP (ng/ml)	**0.009**		0.331	0.053		
≤300		1.231 (0.810–1.873)				
>300						
N/A						
CHST4 Expression	**0.013**		**0.001**	**0.026**		**0.027**
Low		0.492 (0.318–0.762)			0.677 (0.478–0.958)	
High						

*HBV, hepatitis B virus; HCC, hepatocellular carcinoma; ALT, alanine transaminase; BCLC, Barcelona Clinic Liver Cancer; AFP, α-fetoprotein; HR, hazard ratio; CI, confidence interval; N/A, not available. Bold values means that the results are statistically significant.*

### CHST4 Inhibits Proliferation and Migration of HCC Cells

**FIGURE 5:**
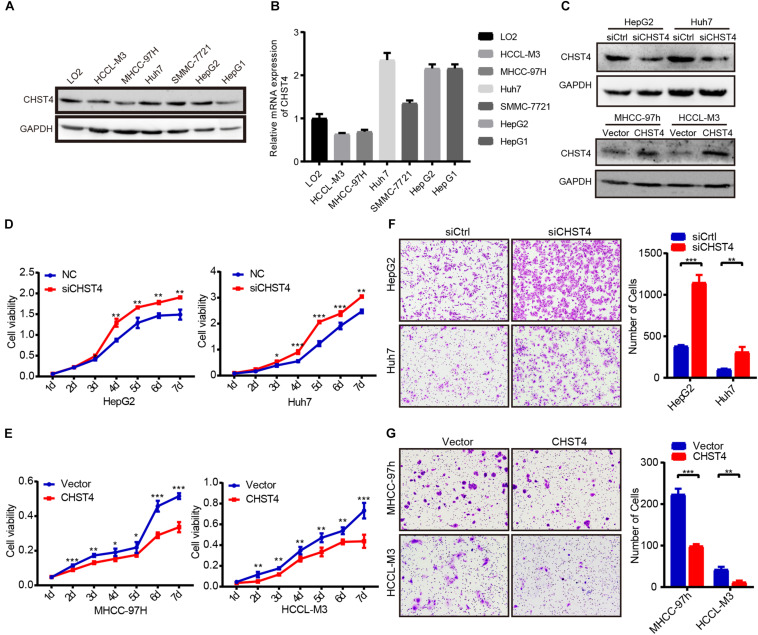
CHST4 inhibits the proliferation and migration of HCC cells. **(A,B)** Protein and mRNA expression of CHST4 in various HCC cell lines. **(C)** Knockdown efficiencies were validated in HepG2 and Huh7 cells, and overexpression efficiencies were validated in MHCC-97h and MCCL-M3 cells by Western blot. **(D,E)** Effects of CHST4 knockdown and overexpression on HCC proliferation determined by CCK-8 assays. Bars represent the mean ± SD; *P < 0.05, **P < 0.01, ***P < 0.001 (Student’s *t*-test).588896105588896105 **(F,G)** Effects of CHST4 knockdown and overexpression on HCC migration were determined by Transwell assays. The right panels show the respective quantitative histograms. All experiments were performed in triplicate. Bars represent the mean ± SD; *P < 0.05, **P < 0.01, ***P < 0.001 (Student’s *t*-test).588896108588896108.

As low CHST4 expression in HBV-HCC tumor tissues was associated with malignant clinicopathological features, we explored the biological function of CHST4 in HCC cells. First, we examined the CHST4 expression pattern in HCC cell lines (HCCL-M3, MHCC-97h, Huh7, SMMC-7721, hepG2, and HepG1) and normal liver cells (L02) (Figures 5A,B). Next, according to the endogenous level of CHST4, we silenced CHST4 in HepG2 and Huh7 cells using a CHST4 siRNA and overexpressed CHST4 in MHCC-97h and HCCL-M3 cell lines using a CHST4 plasmid. The efficiencies of CHST4 knockdown and overexpression were validated by qRT-PCR and Western blot (Figure 5C and Supplementary Figures S6A,B): siRNA2 targeting of CHST4 showed satisfactory knockdown efficiency and was used in the subsequent *in vitro* experiments (Supplementary Figure S6C).

We then investigated the role of CHST4 in the pathogenesis of HCC *in vitro*. CHST4 knockdown resulted in elevated cell viability in HepG2 and Huh7 cells than controls. Conversely, CHST4-overexpressing MHCC-97h and HCCL-M3 cells showed decreased cell viability compared to in control cells (all *P* < 0.05) Figures 5D,E). Transwell migration assays showed that CHST4 knockdown greatly increased the migration abilities of HepG2 and Huh7 cells (Figure 5F). In contrast, CHST4 overexpression hindered the migration capacities of HCC cells (Figure 5G). Together, our data demonstrate that CHST4 is critical for the proliferation and migration of HCC cells.

### FENDRR Inhibits miR-10b-5p Expression to Increase CHST4 Levels in HBV-HCC

There are currently three known regulatory gene expression pathways: DNA methylation (40), RNA splicing (41), and translation blocking (42). Previous studies showed that the promoter of CHST4 is hypermethylated in HBV-HCC (26), which may lead to the downregulation of CHST4 expression. However, intracellular miRNA may also regulate the expression of CHST4 through translation inhibition. Therefore, to explore the upstream regulation of CHST4 expression in HBV-HCC, we constructed a ceRNA network (43).

**FIGURE 6:**
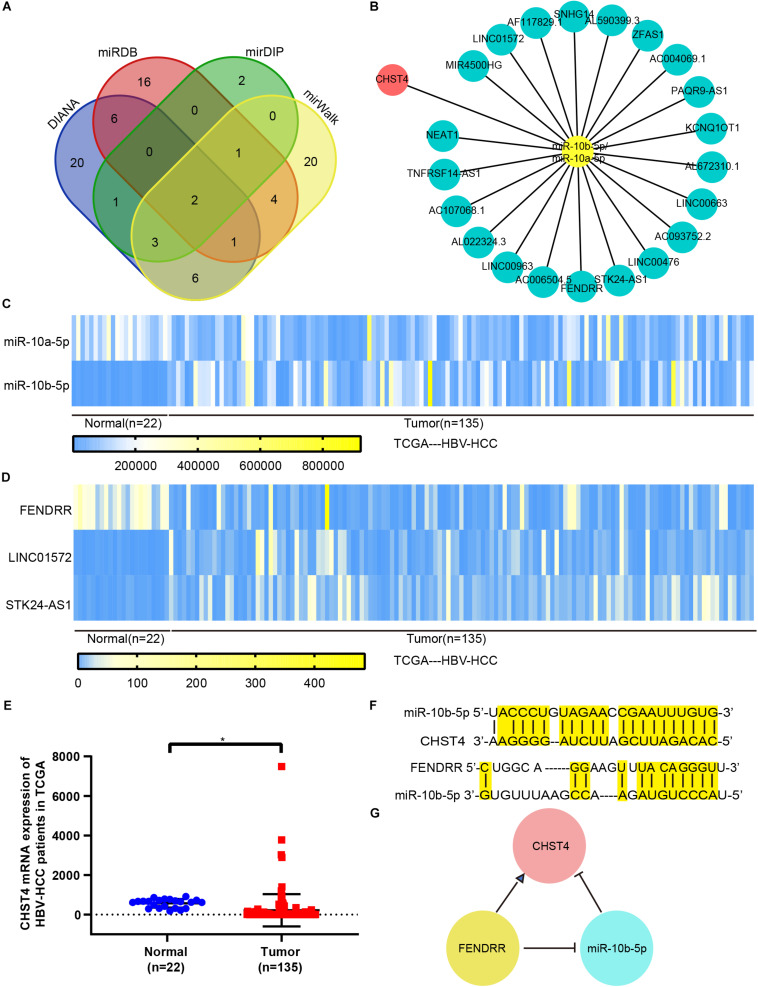
The competing endogenous RNA (ceRNA) network for CHST4 in HBV-HCC. **(A)** Results of the predicted miRNAs using four different databases: miR-10b-5p and miR-10a-5p were finally screened. **(B)** Construction of the lncRNA–miRNA–CHST4 network. **(C–E)** The expression of mRNA, miRNA, and lncRNA in the network of HBV-HCC patients from The Cancer Genome Atlas. Bar represents the mean ± SD; *P < 0.05 (Student’s *t*-test).588896110588896110 **(F)** Complementary sequences between CHST4 and miR-10b-5p, and miR-10b-5p with FENDRR. **(G)** A ceRNA network constructed using FENDRR, miR-10b-5p, and CHST4 in HBV-HCC.

Using the DIANA, miRDB, mirDIP, and miRwalk databases to predict upstream miRNAs of CHST4, we identified miR-10b-5p and miR-10a-5p as potential regulators (Figure 6A). As miR-10a-5p shares the same seed sequence with miR-10b-5p, they may target the same lncRNAs. Therefore, we used DIANA LncBase V2 to predict the lncRNAs that are targeted by these miRNAs in the liver and constructed a potential lncRNA–miRNA–CHST4 ceRNA network (Figure 6B).

We compared the expression levels of CHST4 and differentially expressed miRNAs and lncRNAs between normal and tumor tissues in human HBV-HCC from TCGA. We screened HBV-HCC patients (i.e., those who were positive for the hepatitis B surface antigen) with complete clinical data in TCGA, and identified 135 HBV-HCC tumor tissues and 22 normal liver tissues. CHST4 expression was significantly lower in HBV-HCC tumor tissues than the corresponding normal tissue, while only miR-10b-5p was more highly expressed (*P* < 0.01) (Figures 6C,E). Therefore, miR-10b-5p is likely the miRNA target of CHST4 in HBV-HCC. We also found that the lncRNA FENDRR was highly expressed in tumors (*P* < 0.01) (Figure 6D). Therefore, we analyzed any complementary sequences between CHST4 and miR-10b-5p, miR-10b-5p and FENDRR, respectively (Figure 6F). Together, our findings suggest that the lncRNA FENDRR inhibits the expression of miR-10b-5p, which, in turn, increases the expression of CHST4 in HBV-HCC (Figure 6G).

### CHST4 May Recruit Immune Cells to the Tumor Microenvironment

**FIGURE 7:**
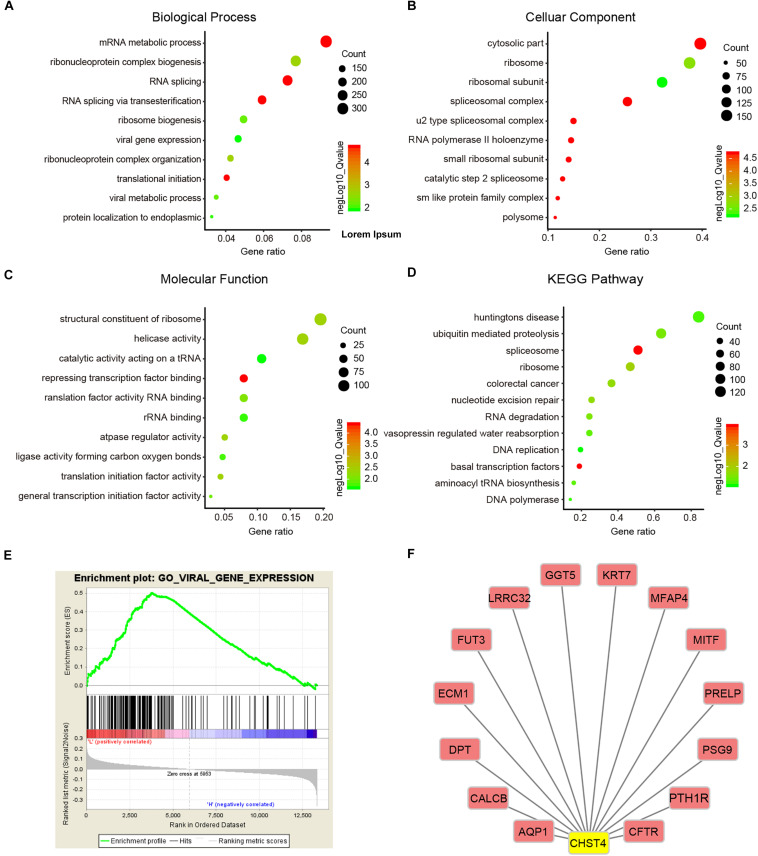
Gene function and downstream pathway analysis for CHST4. **(A–D)** Gene ontology (GO) analysis of CHST4 for HBV-HCC patients in the GSEA14520 dataset by gene set enrichment analysis (GSEA). **(E)** Relationship between CHST4 and viral gene expression by GO analysis. **(F)** The co-expressed genes of CHST4 in GSE14520.

Next, we performed GSEA to investigate the potential biological roles of CHST4 using the Molecular Signatures Database (MSigDB) for GSE14520. GO analysis showed that CHST4 participates in ribonucleoprotein complex biogenesis, RNA splicing, and mRNA metabolic process, and was also closely related to viral gene expression (Figures 7A–C,E). Furthermore, the KEGG pathway analysis showed that CHST4 regulates the ubiquitin-mediated proteolysis signaling pathway and takes part in nucleotide excision repair, DNA replication, and aminoacyl tRNA biosynthesis (Figure 7D). We also identified the co-expressed genes of CHST4 in GSE14520 (Figure 7F and Supplementary Table S2).

We then constructed a protein–protein interaction (PPI) network to identify 40 significant interacting proteins of CHST4 from the String database (Supplementary Figure S7A) and used the DAVID database to explore the potential roles of CHST4 and these interacting proteins. GO analysis showed these genes were mainly located in the Golgi, several biofilms, and extracellular exosomes. The genes participate in multiple biological metabolic processes, such as sulfur compound metabolism, glycoprotein biosynthesis, and oligosaccharide metabolism. They also regulate the immune response, as well as leukocyte migration, tethering, or rolling (Supplementary Figures S7B–D). The KEGG analysis showed that these genes regulate metabolic pathways and proteoglycans in cancer. They are also involved in the interleukin (IL)-17 signaling pathway and the intestinal immune network for IgA production, and are related to the hematopoietic cell lineage (Supplementary Figure S7E), which indicates that CHST4 may participate in immune regulation of the tumor microenvironment.

**FIGURE 8:**
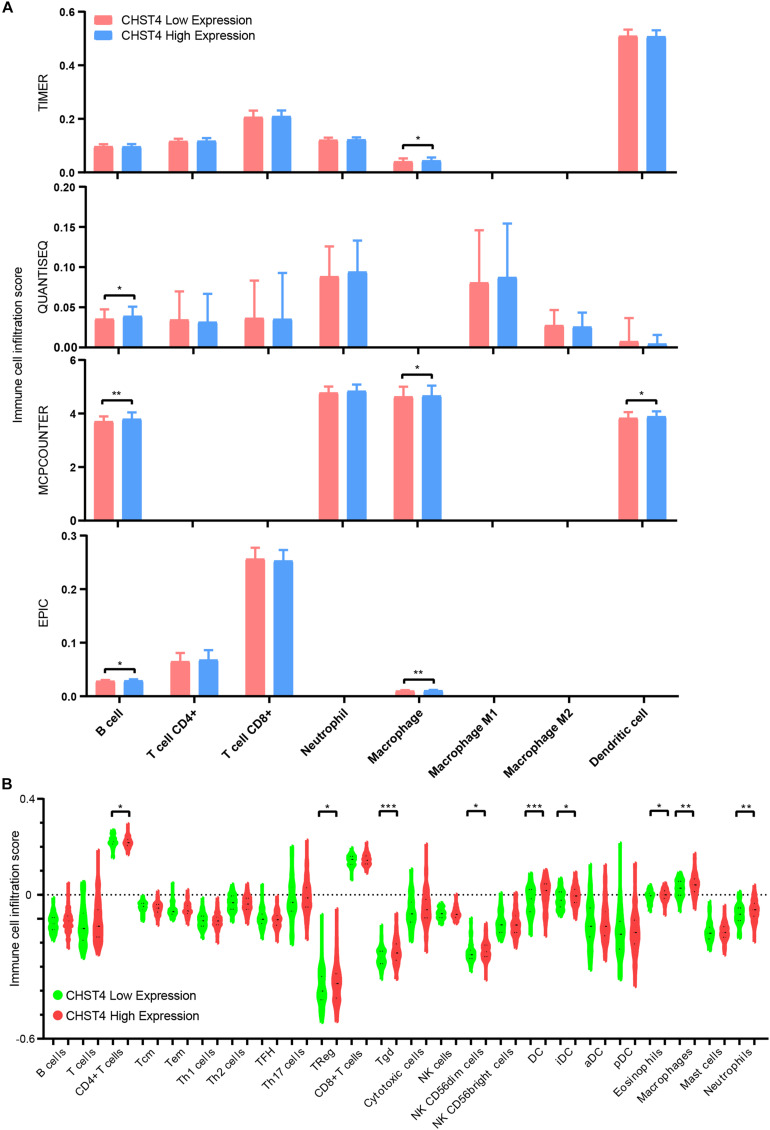
The correlation between CHST4 and immune cell infiltration in HBV-HCC. **(A)** The degree of immune infiltration in HBV-HCC was evaluated by TIMER platform (TIMER, QUANTISEQ, MCPCOUNTER, and EPIC databases). Bar represents the mean ± SD; *P < 0.05, **P < 0.01 (Student’s *t*-test). **(B)** The immune cell infiltration score in HBV-HCC using R-ssGSEA (Tcm, T central memory cells; Tem, T effector memory cells; Th1 cells, T helper 1 cells; Th2 cells, T helper 2 cells; TFH, T follicular helper cells; Th17 cells, T helper 17 cells; Treg, regulatory T cells; Tgd, T gamma delta cells; NK cells, natural killer cells; DC, dendritic cell; iDC, immature DC; aDC, activated DC; pDC, plasmacytoid DC). Bars represent the mean ± SD; *P < 0.05, **P < 0.01, ***P < 0.001 (Student’s *t*-test).

We further evaluated the immune cell infiltration score in HBV-HCC using TIMER platform (TIMER, QUANTISEQ, MCPCOUNTER, and EPIC databases) and R-ssGSEA. We found that those with high expression of CHST4 had higher scores of B cells, CD4+ T cells, macrophages, dendritic cells, and neutrophils (Figures 8A,B). Therefore, overall findings indicate that CHST4 can recruit immune cells into the tumor microenvironment in HBV-HCC.

## Discussion

In this study, we showed that CHST4 is highly conserved in mammals, indicating that it has a potential role in species evolution. We found that both protein and mRNA levels of CHST4 were high in the intestines and the lung in BALB/c mice. Similarly, previous studies showed that CHST4 was highly expressed in the mouse colon (44). We also found that protein and mRNA levels of CHST4 were low in the liver in BALB/c mice, while high mRNA levels and low protein levels were observed in the pancreas. In contrast, the mRNA levels of CHST4 were high in the pancreas and liver in C57BL/6 mice (12), which may be related to the different strains of mice (45). The difference in protein and mRNA levels may be due to translation or posttranslational modifications (46) of CHST4, although this has not yet been verified.

We also analyzed the mRNA levels of CHST4 in 34 different types of human tumors. Consistent with previous studies, we found that the expression of CHST4 mRNA was higher in cholangiocarcinoma (24), colon adenocarcinoma (18), and gastric adenocarcinoma (22), and we partially verified this conclusion immunohistochemically. We also found that tumor expression of CHST4 at the mRNA and protein level is only partially consistent. We speculate this difference is partly because the TIMER data from the TCGA database contains ethnic differences, which could result in tumor heterogeneity. Additionally, our specimen size is small, which could also lead to deviations in tumor characteristics. We also found significant differences in the expression of CHST4 among different subtypes of the same tumor. For example, the expression of CHST4 in BRCA-basal was higher than that in BRCA-Her2 and BRCA-luminal. There was also a higher expression of CHST4 in metastatic in SKCM compared to non-metastatic cancer, and the expression of CHST4 was higher in the HPV-positive subtype of HNSCC than in the HPV-negative one. Similarly, differences in CHST4 expression were previously observed among the various subtypes of colon adenocarcinoma (18) and ovarian cancer (19). Therefore, we speculate that CHST4 may be a potential biomarker for specific tumor subtypes, and this hypothesis should be validated in future studies.

We also showed that CHST4 expression was downregulated in HBV-HCC. Moreover, patients from the GSE14520 dataset of HBV-HCC with higher CHST4 expression had longer OS and DFS. Further analysis showed that CHST4 expression was an independent prognostic indicator among patients with HBV-HCC. Moreover, when we combined CHST4 with three other independent prognostic factors (multinodular, cirrhosis, and BCLC stage), the predictive value for prognosis significantly improved. Thus, potentially, these four factors could be useful survival predictors for patients with HBV-HCC.

Additionally, *in vitro* experiments confirmed that ectopic overexpression of CHST4 inhibited the proliferation and metastasis of HCC, while silencing of CHST4 expression promoted tumor progression. These findings indicate that CHST4 has an important biological function and prognostic value in HBV-HCC. Indeed, previous studies show that CHST4 can flow in the bloodstream in soluble form (21); however, whether CHST4 can be used as a serological indicator of HBV-HCC requires further research. Moreover, as the relationship between the expression level of CHST4 and HBV-HCC prognosis was contradictory to the prognosis of HCC from TCGA, the role of CHST4 in other subtypes of HCC should also be further investigated.

Our analysis of the upstream regulatory network of CHST4 in HBV-HCC revealed both FENDRR and miR-10b-5p are most likely involved. FENDRR has previously been shown to play an important role in tumor suppression. For example, in NSCLC, lncRNA FENDRR was lowly expressed and inhibited tumor cell growth and cisplatin resistance by regulating ABCC10 (3). Also, in colorectal cancer, FENDRR interacts with miRNA-18a-5p to upregulate the expression of growth inhibitor 4, thus inhibiting the proliferation, migration, and invasion of colorectal cancer cells (47). In breast cancer, FENDRR inhibits tumor cell proliferation, promotes cell apoptosis, and is related to good prognosis (48). Furthermore, miR-10b-5p is overexpressed in HCC tissues and is associated with poor prognosis (49). Therefore, based on this data from previous studies (combined with the complementary sequences and expressions of miR-10b-5p, CHST4, and FENDRR in HBV-HCC), we speculate that the FENDRR–miR-10b-5p–CHST4 network plays an important role in HBV-HCC, although further experiments are necessary to prove this hypothesis.

CHST4 expression was found to be related to viral gene expression, and thus, low CHST4 expression may promote HBV expression and replication in HCC, thereby increasing chromosomal instability and tumor cell proliferation, as reported previously (6). We also discovered that CHST4 participates in ribonucleoprotein complex biogenesis, RNA splicing, and mRNA metabolic process, and these biological processes have previously been implicated in HCC development (50, 51). Moreover, ribosome biogenesis and mutations of ribosome genes were previously shown to be related to progression of various tumors (52, 53). CHST4 may also regulate nucleotide excision repair, which is involved in every step of the DNA recognition–unwinding–incision process, which affects HCC risk (54). We also found that downregulation of CHST4 may impact the immune response and the IL-17 signaling pathway. IL-17 recruits neutrophils to the peritumor matrix of HCC to produce matrix metalloproteinase-9, which stimulates angiogenesis (55). Overall, these findings suggest that the low expression of CHST4 in HBV-HCC may upregulate HBV expression and regulate spliceosome and ribonucleoprotein complex biogenesis to promote malignant behaviors.

Finally, we showed that CHST4 expression positively correlates with the score of infiltration of B cells, CD4 + T cells (56), macrophages (57, 58), dendritic cells (59), and neutrophils (60–62). Therefore, CHST4 might recruit immune cells with tumor-killing activity into the tumor microenvironment, thus inhibiting the occurrence and development of HBV-HCC.

## Conclusion

Our study provides evidence that CHST4 expression may relate to tumor heterogeneity in several types of cancers. In particular, we showed that CHST4 inhibits HCC progression, likely through recruiting immune cells to the tumor microenvironment. We also showed that CHST4 is a prognostic candidate for HBV-HCC, particularly if its expression is combined with multinodular, cirrhosis, and BCLC stage. The function and mechanism of CHST4 in various tumors and their subtypes, and its role as a potential biomarker, should be further explored.

## Data Availability Statement

The original contributions presented in the study are included in the article/Supplementary Material, further inquiries can be directed to the corresponding author/s.

## Ethics Statement

The studies involving human participants were reviewed and approved by the Institutional Review Board of Nanfang hospital. The patients/participants provided their written informed consent to participate in this study. The animal study was reviewed and approved by Institutional Animal Care Committee of Southern Medical University.

## Author Contributions

JG and LC contributed to the study design and draft revision. LZ completed most of the cytologic experiments, drafted the manuscript, and coordinated the data collection and analysis. YF and XWa contributed to data collection and interpretation of bioinformatics results. JL, MY, and XWu contributed to the human specimen collection and analyzed the clinicopathological characteristics of the patients. WenH, LL, and WeiH contributed to implementation and analysis of qRT-PCR and immunohistochemical assay. LY, HP, and YW contributed to mice and tissue preparation. All authors contributed to the article and approved the submitted version.

## Conflict of Interest

The authors declare that the research was conducted in the absence of any commercial or financial relationships that could be construed as a potential conflict of interest.

## Footnotes

1 https://www.genecards.org

2 https://www.uniprot.org

3 http://cistrome.dfci.harvard.edu/TIMER

4 https://portals.broadinstitute.org/ccle

5 https://www.cbioportal.org

6 http://gepia.cancer-pku.cn

7 http://www.ehbio.com/ImageGP

8 http://string-db.org

9 https://portal.gdc.cancer.gov

10 https://www.ncbi.nlm.nih.gov/geo
