# Supplementary material for: Carbohydrate Sulfotransferase 4 Inhibits the Progression of Hepatitis B Virus-Related Hepatocellular Carcinoma and Is a Potential Prognostic Marker in Several Tumors
Source: Front Oncol. 2020 Oct 15;10:554331. doi: 10.3389/fonc.2020.554331 (PMC7593664; doi:10.3389/fonc.2020.554331)
Supplement: Supplementary file 1 [file Data_Sheet_1.docx]

Supplementary Material (8 supplementary figures and 2 supplementary table)

1. Supplementary Figures


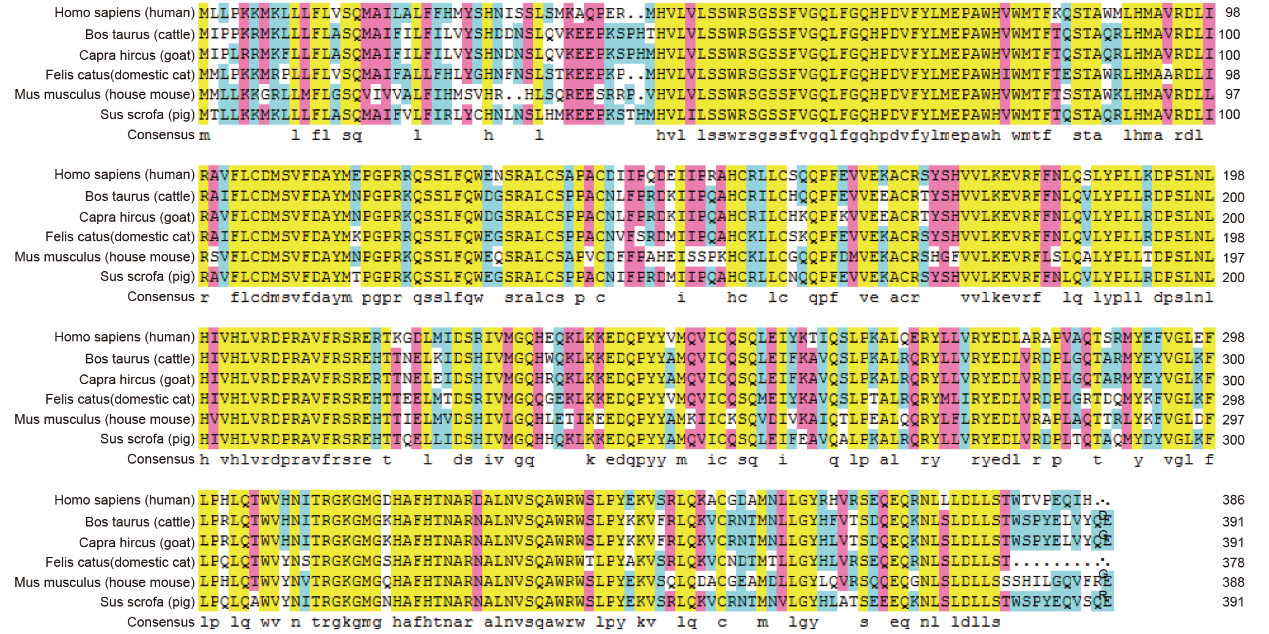


**Figure S1. Alignments analysis of CHST4 protein sequence among Homo sapiens, Bos Taurus, Capra hircus, Felis catus, Mus musculus and Sus scrofa.**


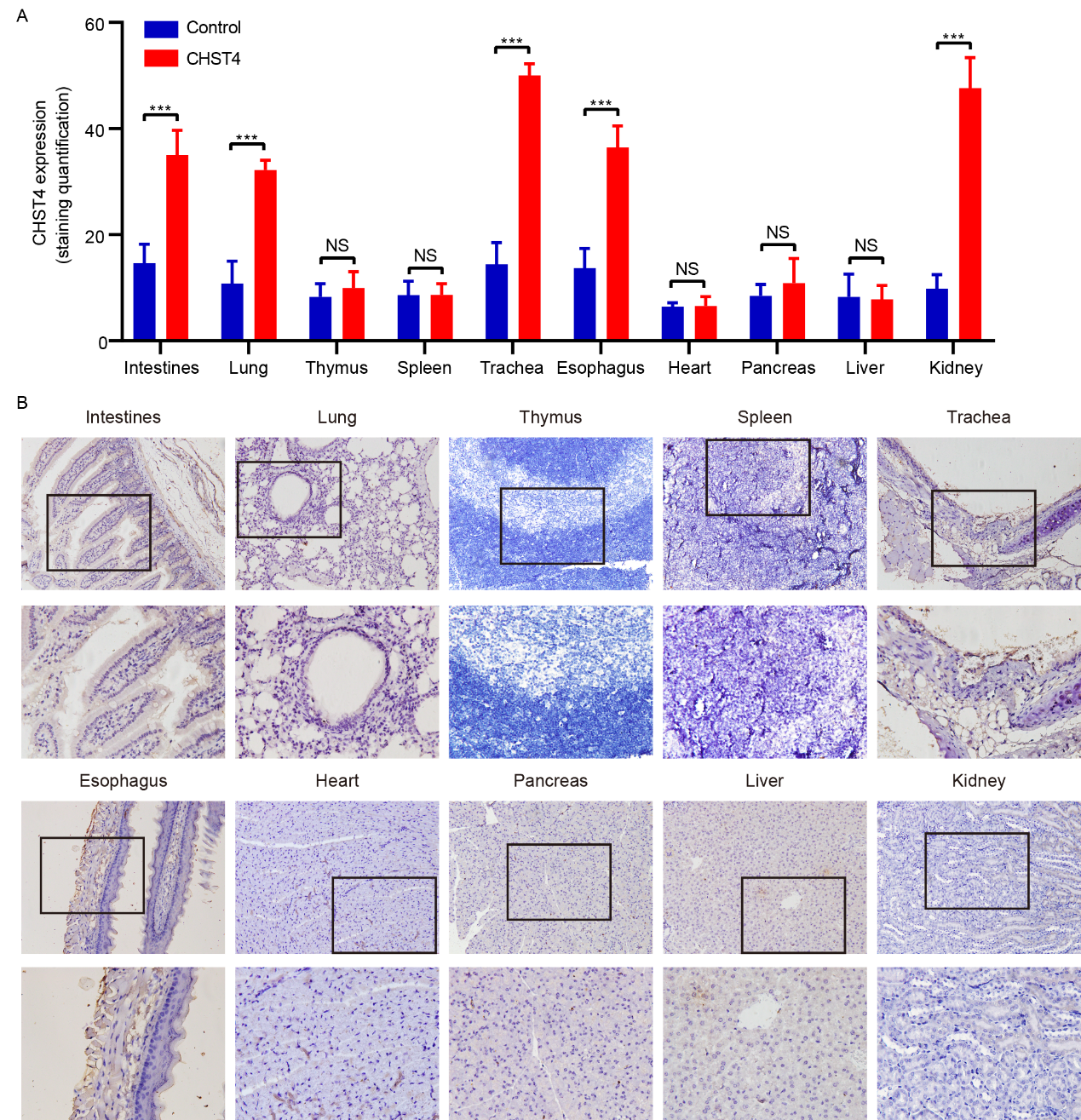


**Figure S2. Quantitative expression of CHST4 staining with ImageJ software and** **the corresponding negative control of immunohistochemical analysis of the expression and distribution of CHST4 protein in BALB/c mice tissues.** **(A)** Quantitative expression of CHST4 staining with ImageJ software. Bars represent the mean ± SD (four samples in each group with five fields per tissue); ***P <0.001 (student’s t-test), NS, not significant. **(B)** The corresponding negative control of immunohistochemical analysis of the expression and distribution of CHST4 protein in BALB/c mice tissues.


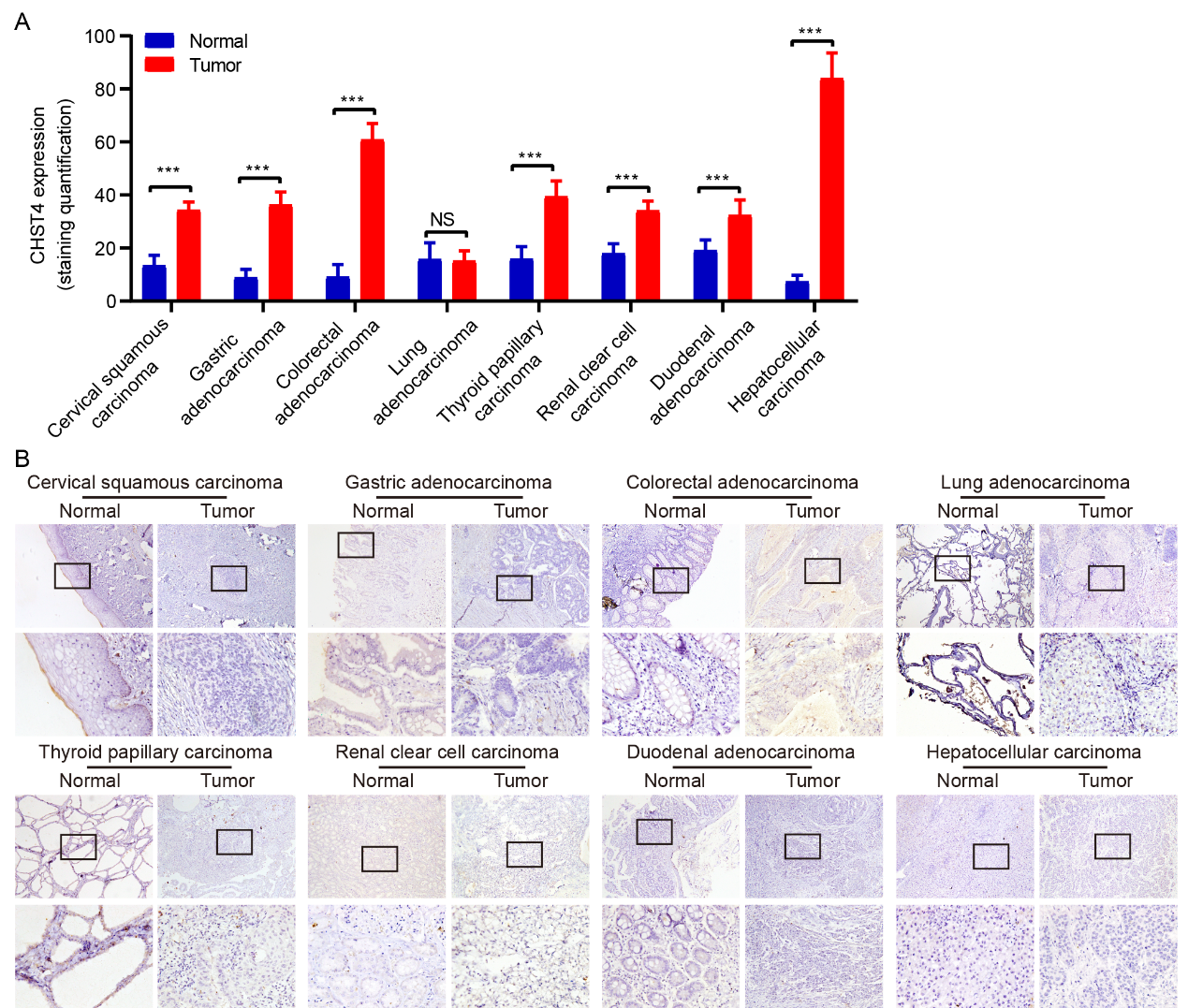


**Figure S3. Quantitative expression of CHST4 staining with ImageJ software and the corresponding negative control of immunohistochemical analysis of the expression and distribution of CHST4 protein in human tissues. (A)** Quantitative expression of CHST4 staining with ImageJ software. Bars represent the mean ± SD (more than three samples in each group with five fields per tissue); ***P <0.001 (student’s t-test), NS, not significant. (B) The corresponding negative control of immunohistochemical analysis of the expression and distribution of CHST4 protein in human tissues.


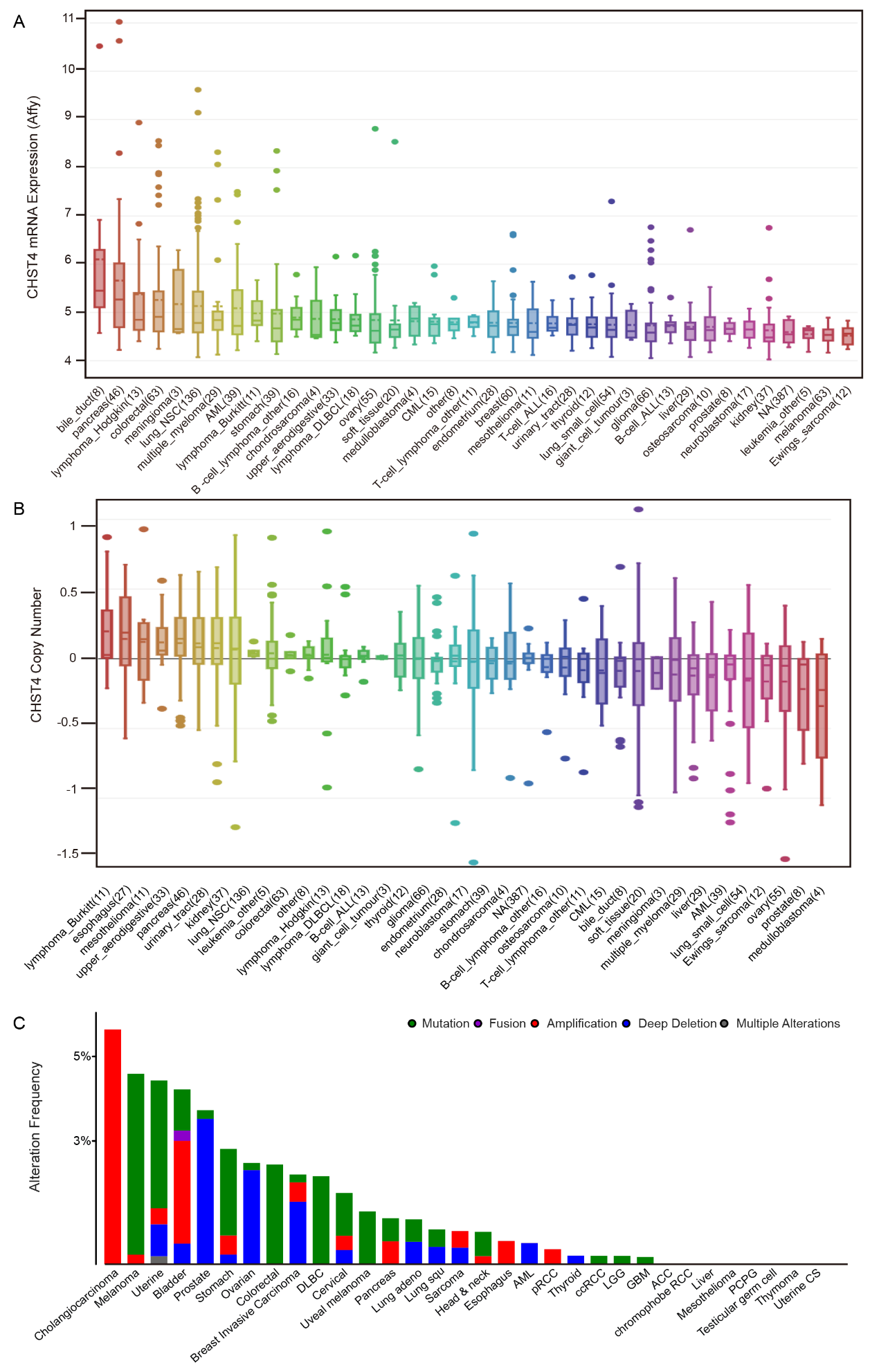


**Figure S4. Expression and Copy number of CHST4 in human cancer cell lines and gene altered in human tumors. (A-B)** Expression and Copy number of CHST4 in human cancer cell lines analyzed by CCLE database. **(C)** The gene altered of CHST4 in 32 human tumor types from cBioPortal database.


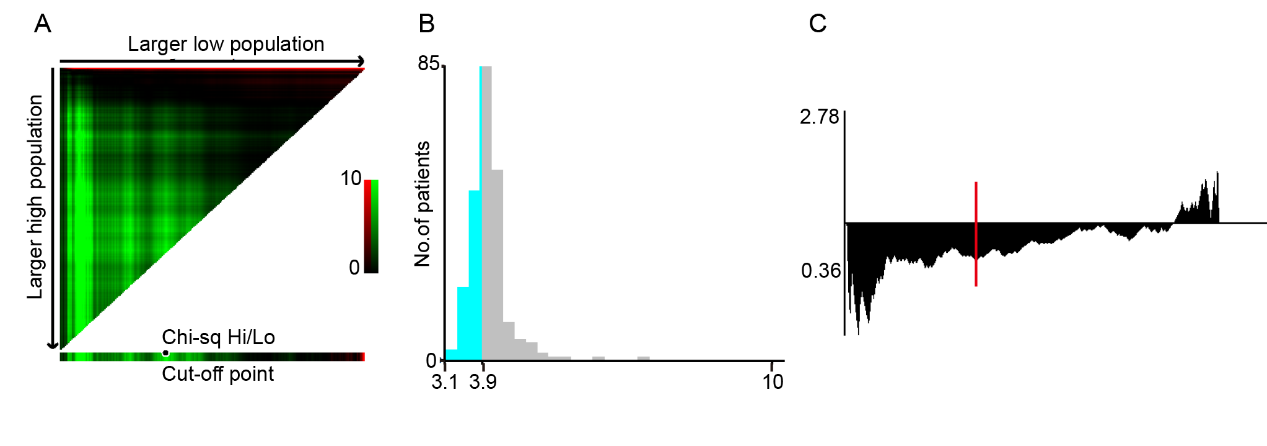


**Figure S5. The x-tile analysis demonstrating the optimal cutoff values of CHST4 expression in tumor tissues of GSE14520.** **(A)** X-tile plots based on the expression of CHST4. The data on the horizontal ordinate increase from the left to the right, defined as the large low population. The data on the vertical ordinate increase from the top to the bottom, defined as the large high population. **(B)** The optimal cutoff values of CHST4 expression. **(C)** Relative risk plot of patients with high CHST4 expression compared with those with low expression.


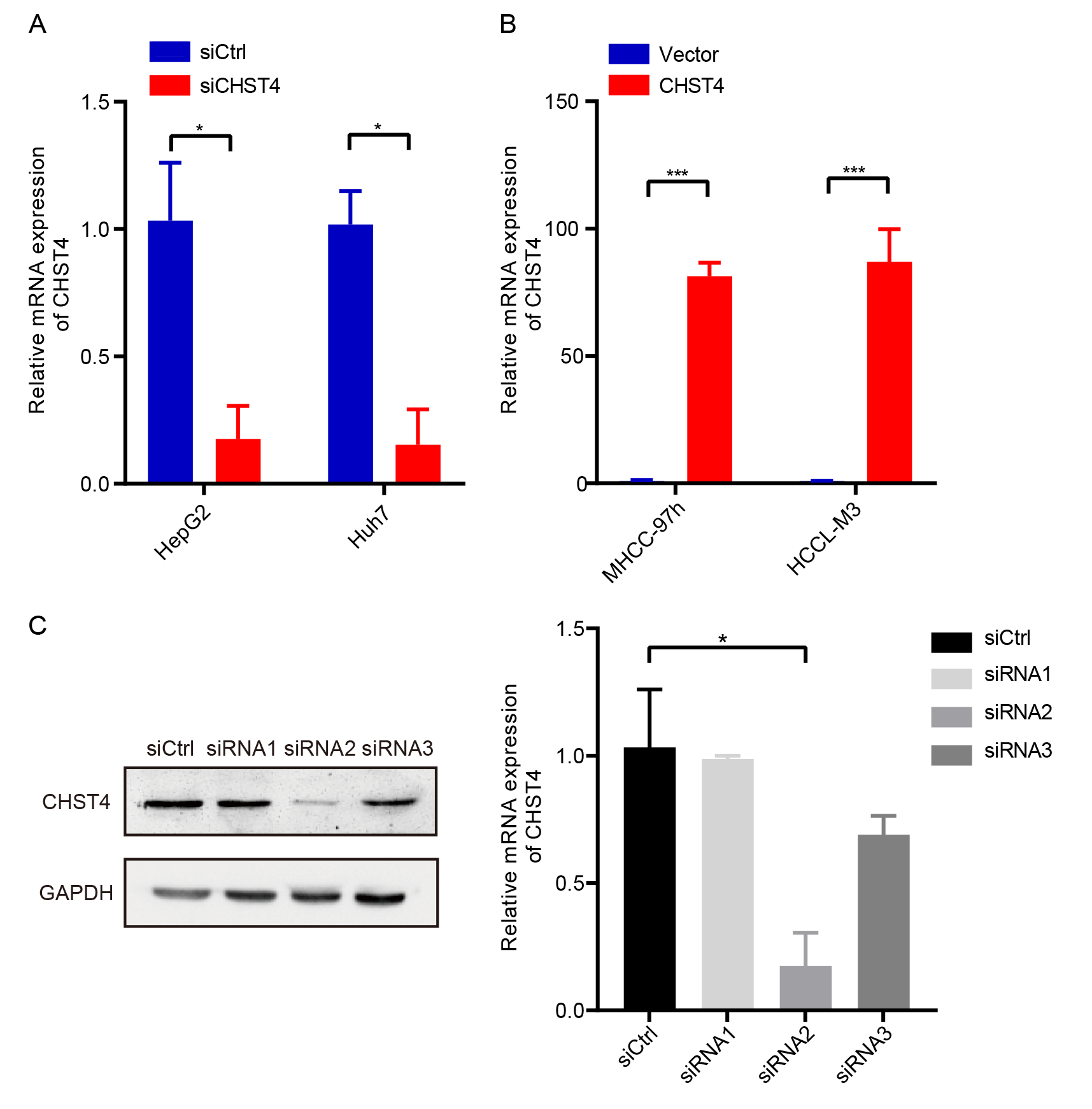


**Figure S6. Verification of siRNA & plasmid transfection efficiency of CHST4. (A-B)** Knockdown efficiencies were validated in HepG2 and Huh7 cells and overexpression efficiencies were validated in MHCC-97h and MCCL-M3 cells by qRT-PCR assays. All samples were tested in triplicate. Bars represent the mean ± SD; *P <0.05, **P <0.01, ***P <0.001 (student’s t-test). **(C)** The efficiencies of CHST4 siRNAs were validated by western blotting and qRT-PCR assays. All samples were tested in triplicate. Bars represent the mean ± SD; *P <0.05, **P <0.01, ***P <0.001 (student’s t-test).


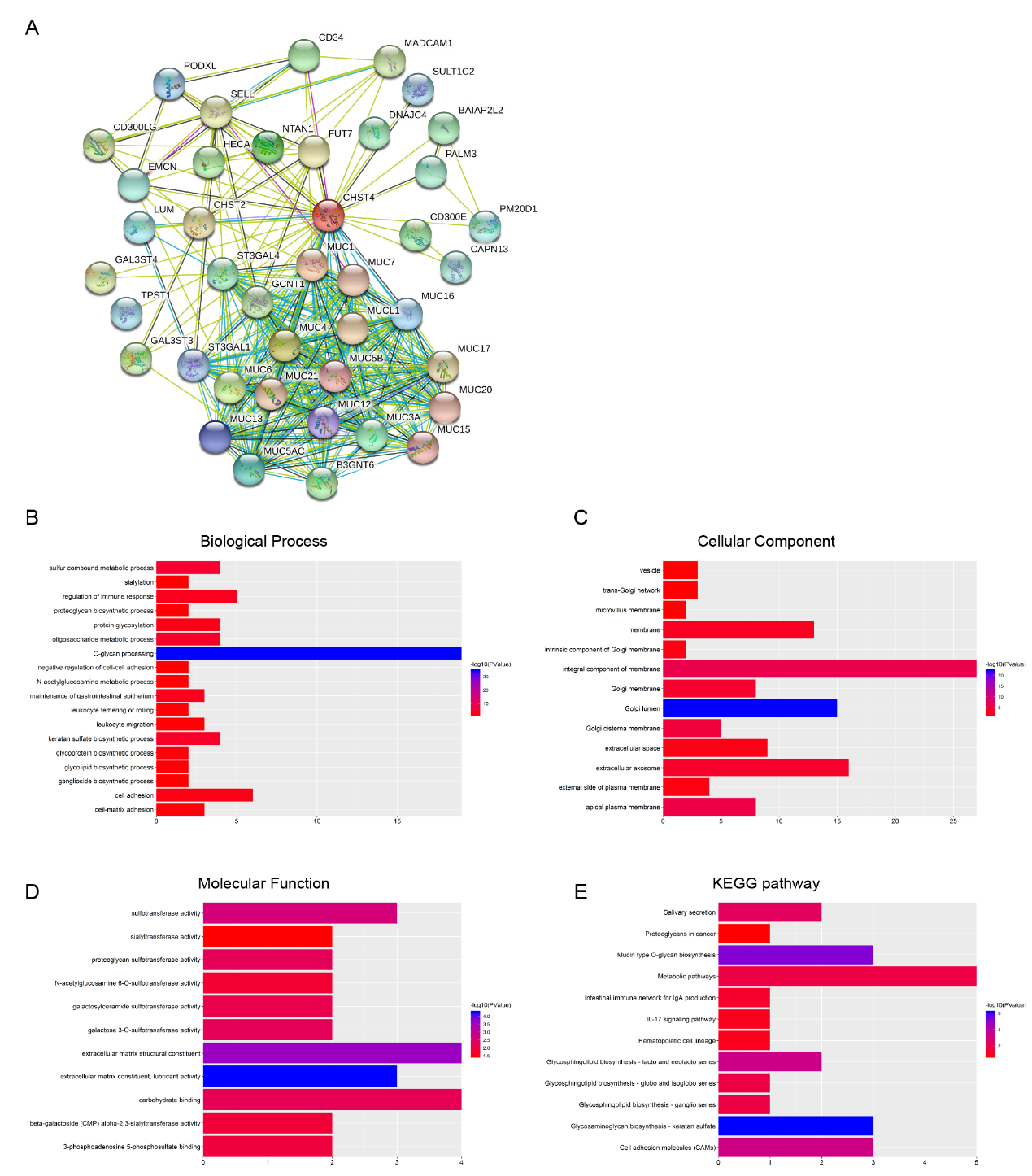


**Figure S7. GO and KEGG pathway enrichment analysis for Interacting proteins of CHST4 form String database. (A)** A PPI network for interacting proteins of CHST4 by String database. **(B-D)** GO analysis for interacting proteins of CHST4. **(B)** biological process, **(C)** cellular component, **(D)** molecular function. **(E)** KEGG pathway enrichment analysis of CHST4 interacting genes.

2. Supplementary Tables

**Table S1. The AUC (the area under the curve) of ROC curve for clinicopathological risk factors of patients in GSE14520.**

| **Variables** | **AUC** | **P** | **95% CI** |
| --- | --- | --- | --- |
| CHST4 | 0.588 | 0.027 | 0.51-0.665 |
| BCLC stage | 0.639 | <0.001 | 0.562-0.716 |
| BCLC+2 factors ^§^ | 0.673 | <0.002 | 0.599-0.746 |
| 4 factors ^ψ^ | 0.72 | <0.003 | 0.651-0.79 |

Notes: ^§^BCLC stage and 2 factors (multinodular and cirrhosis); ^ψ^4 factors (CHST4 expression, BCLC stage, multinodular and cirrhosis)

**Table S2. The co-expressing genes of CHST4 in GSE14520.**

| **Gene1** | **Gene2** | **r** | **P-value** |
| --- | --- | --- | --- |
| CHST4 | AQP1 | 0.449 | 2.22E-13 |
| CHST4 | CALCB | 0.472 | 7.92E-15 |
| CHST4 | CFTR | 0.424 | 5.91E-12 |
| CHST4 | DPT | 0.444 | 4.06E-13 |
| CHST4 | ECM1 | 0.47 | 1.00E-14 |
| CHST4 | FUT3 | 0.404 | 6.32E-11 |
| CHST4 | LRRC32 | 0.402 | 7.80E-11 |
| CHST4 | GGT5 | 0.447 | 2.92E-13 |
| CHST4 | KRT7 | 0.401 | 9.17E-11 |
| CHST4 | MFAP4 | 0.443 | 4.56E-13 |
| CHST4 | MITF | 0.412 | 2.34E-11 |
| CHST4 | PRELP | 0.491 | 4.37E-16 |
| CHST4 | PSG9 | 0.453 | 1.20E-13 |
| CHST4 | PTH1R | 0.437 | 1.01E-12 |

Notes: r, two-side Pearson correlation coefficient. P<0.05 was considered statistically significant (z-test).
